# RhlR-mediated cooperation in cystic fibrosis-adapted isolates of *Pseudomonas aeruginosa*

**DOI:** 10.1128/jb.00344-24

**Published:** 2024-12-13

**Authors:** Renae L. Cruz, Tiia S. Freeman, Kyle L. Asfahl, Nicole E. Smalley, Ajai A. Dandekar

**Affiliations:** 1 Department of Microbiology, University of Washington 312771 , Seattle, Washington, USA; 2 Department of Medicine, University of Washington 205280 , Seattle, Washington, USA; University of Illinois Chicago, Chicago, Illinois, USA

**Keywords:** cooperation, social dynamics, RhlR, quorum sensing

## Abstract

*Pseudomonas aeruginosa* uses quorum sensing (QS) to regulate the expression of dozens of genes, many of which encode shared products, called “public goods.” *P. aeruginosa* possesses two complete acyl-homoserine lactone (AHL) QS circuits: the LasR-I and RhlR-I systems. Canonically, these systems are hierarchically organized: RhlR-I activity depends on LasR-I activation. However, in contrast to laboratory strains, isolates from people with cystic fibrosis can engage in AHL QS using only the transcription factor RhlR. In these isolates, RhlR regulates AHL QS and the production of secreted public goods, such as the exoprotease elastase, which are accessible to both producing and non-producing cells. When *P. aeruginosa* strains that use LasR to regulate elastase production are grown on casein as the sole carbon and energy source, LasR-null mutant “cheaters” commonly arise in populations due to a selective growth advantage. We asked if these social dynamics might differ in “RhlR cooperators”: populations that use RhlR, not LasR, to regulate public goods. We passaged RhlR cooperators from several genetic backgrounds in casein broth. We found that cheaters emerged among most RhlR cooperators. However, in one isolate background, E90, RhlR-null mutants were dramatically outcompeted by RhlR cooperators. In this background, the mechanism by which RhlR mutants are outcompeted by RhlR cooperators is AHL-dependent and occurs in stationary phase but is not the same as previously described “policing” mechanisms. Our data suggest that cheating, or the lack thereof, does not explain the lack of RhlR mutants observed in most infection environments.

**IMPORTANCE:**

Quorum sensing (QS) mutants arise in a variety of populations of bacteria, but mutants of the gene encoding the transcription factor RhlR in *Pseudomonas aeruginosa* appear to be infrequent. Our work provides insight on the mechanisms through which RhlR-mediated cooperation is maintained in a LasR-null population of *P. aeruginosa*. Characterizing the selective pressure(s) that disfavor mutations from occurring in RhlR may enhance our understanding of *P. aeruginosa* evolution in chronic infections and potentially guide the development of therapeutics targeting the RhlR-I QS circuit.

## INTRODUCTION

Cooperation, a social behavior that benefits other members of a population, is ubiquitous in the natural world. In bacterial populations, an example of cooperation is the production of public goods such as extracellular proteases and toxins, which are metabolically costly for individual cells to produce but can be utilized by all members of the group. Populations of cooperators are susceptible to invasion by defectors or “cheaters,” cells that forgo the metabolic burden associated with cooperation but still benefit from the public goods produced by cooperators (1). For this reason, cooperation is potentially unsustainable, and its maintenance in populations is widely studied by evolutionary biologists (2).

*Pseudomonas aeruginosa*, a common environmental Gram-negative bacterium, has been used as a model organism for studying cooperation and conflict in bacterial populations (3, 4). Several of its potentially cooperative behaviors are regulated by quorum sensing (QS), a cell-cell communication system mediated by small signaling molecules. *P. aeruginosa* QS is complex and consists of two complete acyl-homoserine lactone (AHL) QS circuits: LasR-LasI and RhlR-RhlI (5). LasR recognizes the signal *N*-3-oxo-dodecanoyl homoserine lactone (3OC12-HSL), which is produced by the signal synthase LasI; RhlR recognizes the signal *N*-butanoyl homoserine lactone (C4-HSL) produced by RhlI. In the laboratory strain PAO1, these systems have overlapping regulons and are hierarchically arranged such that LasR largely controls the transcription of RhlR and a non-AHL QS transcription factor, PqsR (6, 7). LasR therefore has been considered a master QS regulator in PAO1.

One example of a quorum sensing-regulated cooperative behavior is the production of the exoprotease elastase, which is required for growth using either bovine serum albumin or casein as a sole carbon and energy source (3, 4). Elastase is secreted into the extracellular milieu and proteolyzes casein into constituent amino acids or peptides, which can be readily used by *P. aeruginosa* as a carbon and energy source. In a well-mixed environment, all cells in the population can benefit from elastase production, whether or not they produce it themselves; therefore, elastase constitutes a “public good” (3). Previously, we and others have demonstrated that cheaters can eventually invade populations of quorum sensing-proficient cooperators (4, 8) when *P. aeruginosa* PAO1 is passaged in minimal media supplemented with 1% casein. These cheaters are quorum sensing-deficient and commonly harbor deleterious mutations in *lasR;* however, no experimental evolution studies to date have reported mutations occurring in *rhlR*. Similarly, in surveys of *P. aeruginosa* isolates from the cystic fibrosis (CF) lung, *rhlR* mutations are either rare or only occur decades following mutations in *lasR* (9).

The rarity with which *rhlR* mutations occur may reflect that loss of RhlR activity imparts a competitive disadvantage in co-culture with RhlR-proficient strains (8, 10, 11). Because *rhlR* mutations are uncommon in both experimental evolution studies as well as the CF lung, we hypothesized that a mechanism intrinsic to RhlR QS in *P. aeruginosa* likely maintains this system within the population. One possible mechanism through which RhlR-null mutants are suppressed by RhlR-proficient strains is the RhlR-regulated production of, and resistance to, hydrogen cyanide (HCN) (8, 12). Other potential mechanisms, including the production of phenazines or rhamnolipids by cooperators (13, 14), have also been proposed. In light of recent studies that show LasR-null strains can have an active RhlR QS system independent of LasR regulation (15–17), we were interested in how social dynamics may differ in a population that relies on RhlR instead of LasR to regulate cooperative behavior.

In this work, we studied LasR-null, RhlR-active clinical isolates (15, 18, 19) to assess questions pertaining to RhlR-mediated cooperation. Because these isolates regulate the production of public goods such as elastase using RhlR, they are referred to as “RhlR cooperators.” We asked if RhlR cooperators are subject to cheating, akin to LasR cooperators, and found that in most cases they were; however, in one genetic background, cheaters did not emerge and were, instead, purged from the population. Understanding the contribution of RhlR to the maintenance of cooperation may offer insight on the possible advantages of hierarchical as opposed to parallel arrangement of QS circuits and shed light on the evolution of clinical isolates.

## RESULTS

### Selection of clinical isolates

We have previously described one clinical isolate, E90 (19), that regulates elastase production using RhlR; mutants of this isolate with *rhlR* deleted cannot grow in a minimal medium supplemented with casein (casein broth). We sought to identify a larger pool of such isolates to conduct studies of RhlR cooperation. To do so, we surveyed our cohort of clinical isolates from the Early *Pseudomonas* Infection Control (EPIC) trial (20). We selected six of these isolates to characterize and made *rhlR* deletions in each genetic background (Table S1). We initially asked if the *rhlR* mutants could grow equally well as the parent isolates in either buffered lysogeny broth(LB) or minimal medium supplemented with casamino acids (CAA broth), two conditions where the acquisition of carbon and energy does not depend on RhlR (Fig. S1A and B). We used strain PAO1 and PAO1∆*lasR* as controls, as we have previously used strain PAO1 and the LasR-null variant to dissect features of cooperation and conflict in *P. aeruginosa* populations (8, 21). Several isolates showed large differences in growth yield between the wild-type and the *rhlR-*null mutant, and we excluded these from further analysis. Finally, we asked if the remaining *rhlR* mutants could grow on casein broth (Fig. S1C and D). We found four isolates where the *rhlR* mutant could not grow but the wild type could: E56, E90, E113, and E125. We used these four isolates for subsequent experiments.

### RhlR-null cheaters emerge in most populations of RhlR cooperators

To investigate how social dynamics may differ in a population of RhlR cooperators, we passaged E56, E90, E113, and E125 in casein broth as the sole carbon and energy source for >100 generations, as described in Materials and Methods. When strain PAO1 is serially passaged in casein broth, LasR-null mutants invariably invade the population and reach a frequency of about 20%–30% (4, 21, 22). LasR-null mutants are considered cheaters in this context because they do not produce elastase but still benefit from the production of elastase by QS-proficient cells (3). Under some circumstances, the high frequency of cheaters results in a population collapse by causing a loss of a quorum.

**Fig 1:**
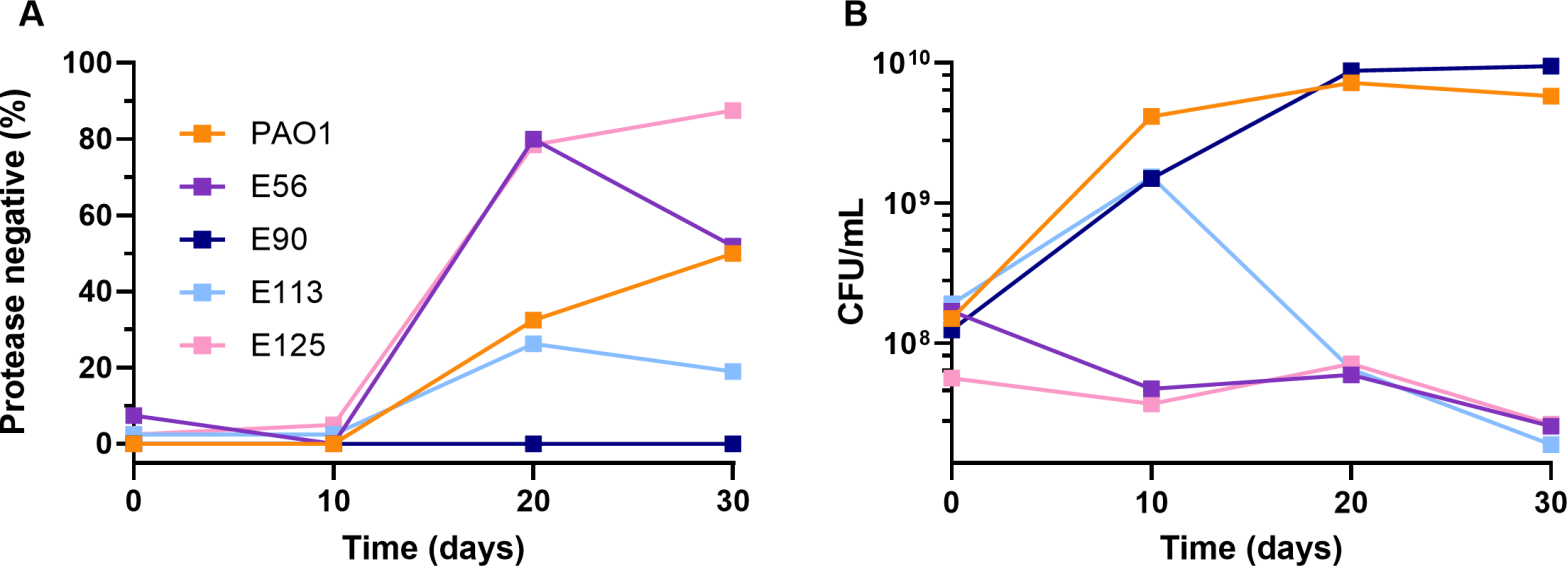
RhlR-null cheaters emerge in most populations of RhlR cooperators. Protease-expressing strains E56, E90, E113, and E125 were passaged in minimal media supplemented with 1% casein for 30 days in an *in vitro* evolution experiment. A representative experiment is shown. At 0, 10, 20, and 30 days, the monoculture populations were assessed for (**A**) frequency isolates with reduced protease production and (**B**) overall bacterial yield. Under the conditions of our experiments, there are ~160 generations of the clinical isolates and ~200 of PAO1.

We predicted that cheaters would also invade the populations of E56, E90, E113, and E125 and that these cheaters would have *rhlR* mutations since RhlR regulates cooperative behaviors including production of elastase in these genetic backgrounds. In three of the four backgrounds (E56, E113, and E125), protease-negative individuals emerged in the population over the 30 days of passage (Fig. 1A). In the case of E56 and E113, these mutants reached a high percentage in the population, and this high percentage was associated with a decrease in total culture productivity, as measured by bacterial yield (Fig. 1B). Because we were interested in the QS regulator RhlR, we sequenced its gene in protease-deficient individuals and found that these isolates all harbored one or more mutations in the coding sequence of *rhlR* (Table 1) consistent with the idea that they were cheating on the wild type by inactivating quorum sensing.

**TABLE 1:** Protease-negative isolates with coding changes, as compared to PAO1, obtained from RhlR cooperator strains passaged in casein medium

Isolate	Parent^*a*^	Timepoint^*b*^	Protease phenotype^*c*^	*rhlR* mutation^*d*^		
				Nucleotide^*e*^	Protein^*f*^	Description^*g*^
4	E113	20	Low	G30A	Trp → *	Nonsense
15	E113	30	None	G30A	Trp → *	Nonsense
19	E125	20	Low	A7G	Asn → Asp	Nonsynonymous
				G239C	Gln → Glu	Nonsynonymous

^*a*^ *P. aeruginosa* clinical isolate parent strain used in casein passaging experiments.

^*b*^ Day of passaging from which the isolate was collected.

^*c*^ Amount of elastase produced relative to the parent strain.

^*d*^ Mutations identified in each isolate from PCR amplification and sequencing of the intergenic region upstream of *rhlR* through the end of the coding sequence of the gene.

^*e*^ Loci of mutations in *rhlR* relative to the coding sequence start in the parent genomic sequence.

^*f*^ Location and change to RhlR protein amino acids, relative to the start codon.

^*g*^ Type of mutation identified.

However, E90 was different: we did not detect the emergence of protease-deficient cheaters after 30 days of passage in five independent trials (Fig. 1A). To exclude the possibility that RhlR-null, constitutive elastase producers invaded the population instead, we performed Sanger sequencing on five protease-positive isolates at the end of the 30-day passage. We found no mutation in either the *rhlR* gene or its promoter sequence. The findings from our experimental evolution studies suggest that *rhlR* mutations are disfavored in the E90 background.

### Mutations in *rhlR* confer a competitive advantage in co-culture, except in E90

**Fig 2:**
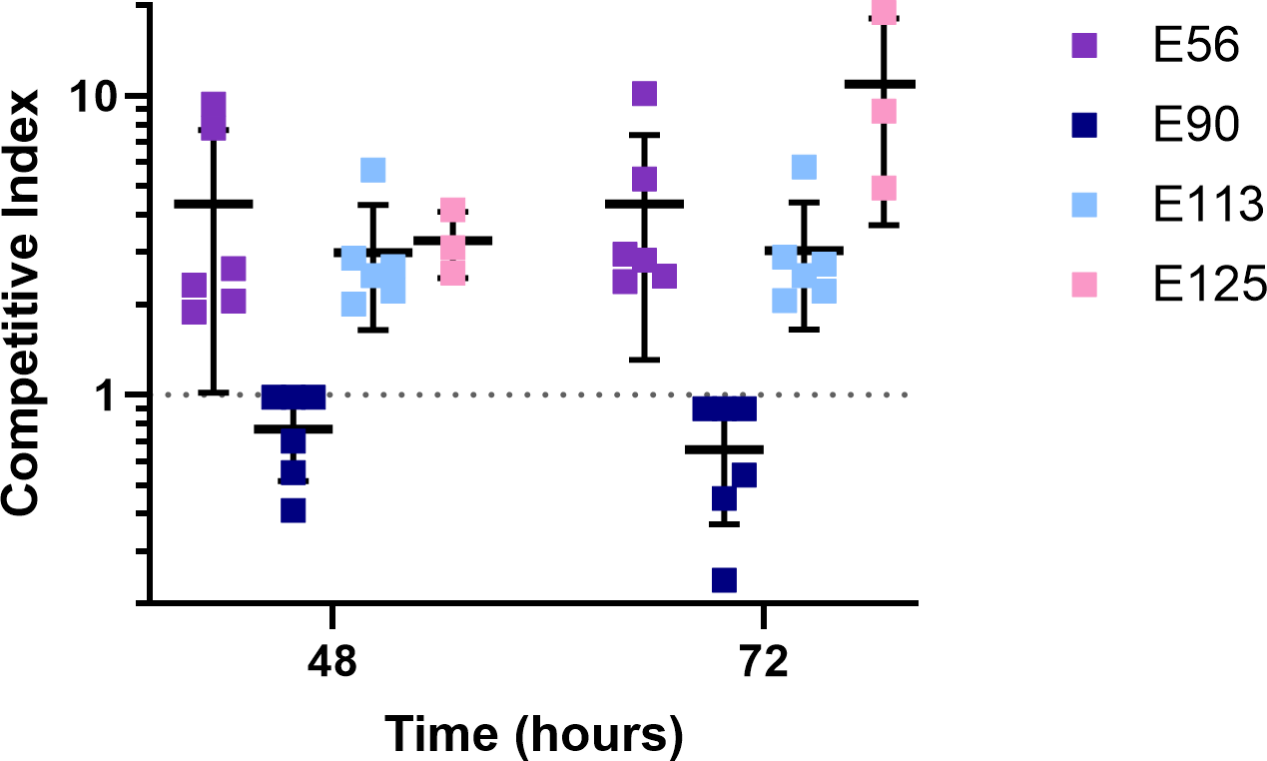
Mutations in *rhlR* confer a competitive advantage in co-culture, except in E90. Green fluorescent protein (GFP)-expressing strains of E56, E90, E113, and E125 RhlR-null mutants were inoculated in co-culture with their parent in 1% casein minimal media at an initial frequency of 10% RhlR-null mutants and passaged every 24 hours. The frequency of fluorescent RhlR-null mutants within the co-culture was assessed via flow cytometry, and a competitive index was determined. In relation to the parent strain, a competitive index of >1 indicates that the strain is more fit, and <1 indicates that it is less fit. Data shown are the average and standard deviation of three biological replicates.

We hypothesized that RhlR mutants invade the population in co-culture because they exhibit a growth advantage compared to the parent strain. To test this idea, we competed E56, E90, E113, and E125 in casein broth against isogenic *rhlR* deletion mutants constructed in each genetic background. We started these competitions with an initial frequency of 10% mutants, based on the fitness advantage of QS mutants observed in previous work (11). Consistent with the results above, we found that the *rhlR* mutants of E56, E113, and E125 increased in frequency rapidly, coming to 50%–70% within 72 hours of inoculation (Fig. 2). For completeness, we competed the full suite of 13 isolates that we initially tested and found that, in most cases, *rhlR* mutants did not noticeably or consistently increase in frequency (Fig. S2), in line with the lack of dependence of growth on QS for many of these strains.

Again, E90 was different from the other strains (Fig. 2). The *rhlR* mutant of E90 grows equally well to the parent in monoculture in minimal media with casamino acids as the sole carbon and energy source (Fig. S3), and therefore, we hypothesized that mutations in *rhlR* imparted a disadvantage in competition. To assess the relative fitness of each strain, we competed an mCherry-tagged E90 RhlR deletion mutant with a CFP-tagged parent. Using flow cytometry to count individual cells, we then calculated the competitive index of the RhlR deletion mutant following 3 days of co-culture with serial passage every 24 hours. We found that the RhlR deletion mutant was substantially less fit than the parent isolate and had a competitive index significantly less than 1 (Fig. 2). Because flow cytometry might enumerate both live and dead cells, we also patched ~100 individual colonies from each co-culture onto skim milk agar plates, as had been done previously (4), to survey the viable population for RhlR mutants. We found that the results from patching onto skim milk agar mirrored the results found by flow cytometry, with a significant reduction in the fraction of cells that were protease-negative (Fig. S4A). Our results suggest that RhlR mutants rarely emerge among RhlR cooperators in the E90 background because loss of RhlR activity confers a competitive defect.

### In E90, RhlR mutants are suppressed through an AHL-regulated mechanism

**Fig 3:**
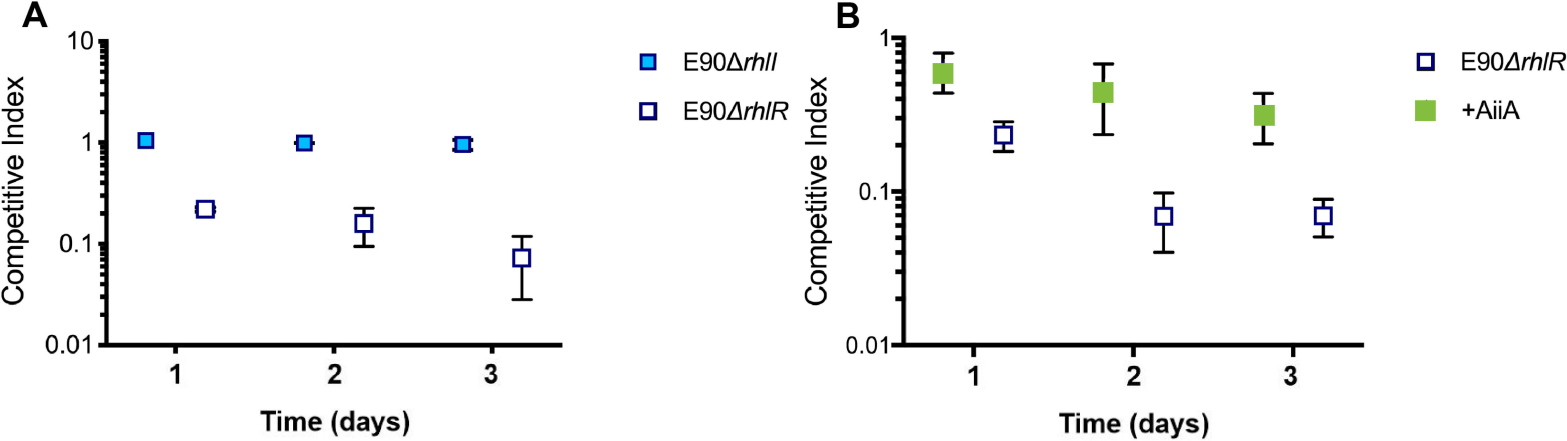
A quorum sensing-regulated factor explains the defect in RhlR-null mutants of E90. (**A**) The competitive index of either the receptor RhlR-null (open boxes) or signal synthase RhlI-null (filled boxes) mutant is displayed on the *y*-axis. (**B**) The addition of AiiA lactonase (filled boxes) rescues the relative fitness of the RhlR-null mutant in co-culture with the parent RhlR cooperator. The competitive index of the RhlR-null mutant following addition of 100 µg/mL AiiA lactonase or a buffer control is displayed on the *y*-axis.

We focused the remainder of our studies on E90, as the cheater-suppressive phenotype we observed in this strain was unexpected. We tested whether the competitive advantage of RhlR cooperators is due to an intact RhlR-RhlI regulatory QS circuit. For this experiment, we co-cultured the parent RhlR cooperator with an isogenic RhlI (C4-HSL synthase) mutant or the RhlR mutant. Both the RhlI and RhlR mutants have an incomplete QS circuit and are incapable of engaging in RhlR QS in monoculture; however, we found that, unlike the RhlR-null mutant, the RhlI mutant was equally fit as the parent strain in co-culture (Fig. 3A). We reasoned that in contrast to the RhlR-null mutant, the RhlR activity of the RhlI mutant is complemented in *trans* during co-culture with the parent strain since C4-HSL is freely diffusible across the membrane. As a complementary approach, we added AiiA lactonase, an enzyme that degrades AHLs (23), to the RhlR cooperator and RhlR mutant co-culture. The addition of lactonase was able to partially rescue the relative fitness of the RhlR mutant (Fig. 3B). Additionally, we found that RhlR mutant growth in monoculture is unperturbed by the addition of 10 µM C4-HSL, the concentration produced by the parent strain, demonstrating that the signal itself is not toxic to RhlR mutants. We concluded that a C4-HSL-RhlR-dependent mechanism confers a competitive advantage to RhlR cooperators, or that degradation of C4-HSL by addition of lactonase resulted in a decreased “cost” to cooperation for cells with intact RhlR QS.

### HCN production and phenazines do not suppress RhlR mutants in the E90 background

**Fig 4:**
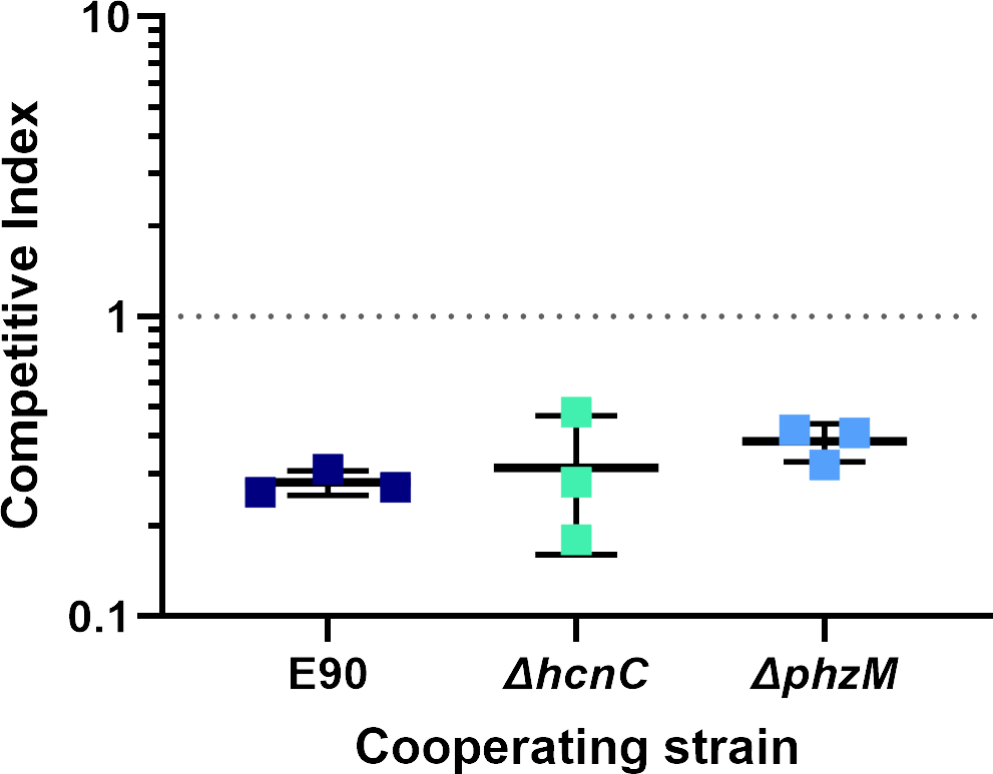
HCN and phenazine production do not control RhlR-null mutants in the E90 background. The competitive index of the RhlR-null mutant following co-culture with the HCN synthase-deficient or the phenazine synthesis-deficient RhlR cooperator is displayed on the *y*-axis. Data shown are the average and standard deviation of three biological replicates.

Previous reports have highlighted the importance of hydrogen cyanide production in “policing” cheaters in QS populations of *P. aeruginosa* (8, 12). In PAO1, the RhlR-regulated production of HCN plays a prominent role in stabilizing cooperation (8); both RhlR mutants and HCN synthase mutants are unable to control LasR-null cheaters in the population. Chen et al. reported similar findings in E80, a LasR-null, RhlR-active *P. aeruginosa* isolate (10). In the latter study, RhlR mutants likely did not emerge in their experiments because RhlR mutants have increased sensitivity to HCN compared to the parent strain. To test whether HCN production was responsible for the suppression of RhlR mutants in the E90 background, we competed an E90 *hcnC* mutant with the E90 *rhlR* mutant and found that the latter was still outcompeted in co-culture (Fig. 4). Our findings suggest that HCN production alone is not responsible for the maintenance of cooperation in E90. This observation is consistent with some other strains of *P. aeruginosa*, which do not appear to produce HCN in concentrations sufficient to police cheaters (24).

Other mechanisms have been implicated in the restraint of cheaters. In structured communities, where cell-cell contact is more extensive than liquid cultures, type VI secretion systems have been implicated in bacterial killing (25). Indeed, the *hsi2* type VI secretion system is RhlR regulated in E90 (19). However, in the well-mixed, shaking conditions of our experiments, there is no evidence that type VI secretion might have a role. Finally, RhlR-regulated phenazines (13) have been suggested to be a mechanism of cheater restraint, but, like HCN, we did not detect a difference in competitive outcomes between a phenazine (*phzM*)-defective cooperator and an RhlR-null mutant (Fig. 4).

### In E90, RhlR-null mutants are outcompeted by RhlR cooperators during stationary phase

**Fig 5:**
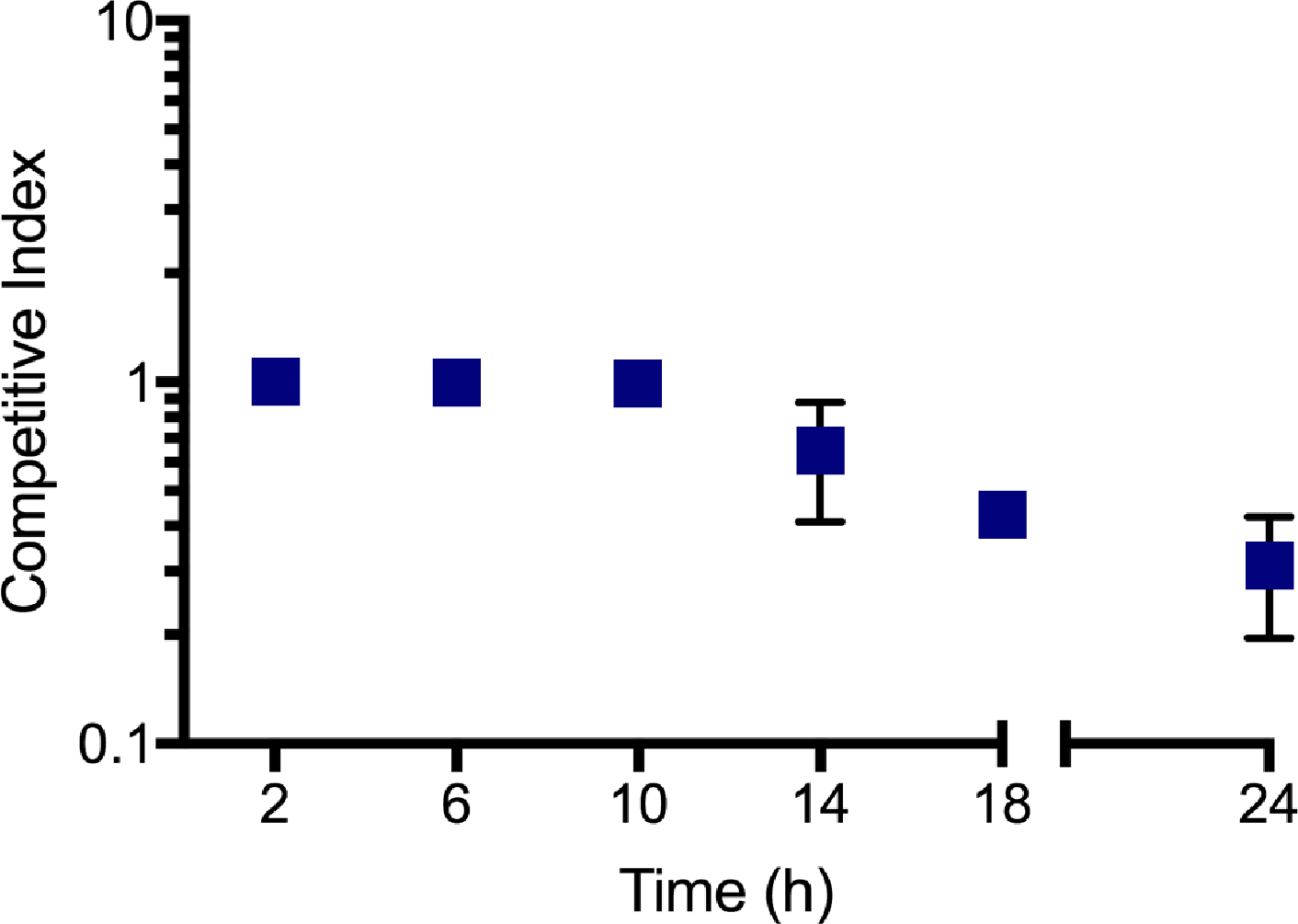
RhlR-null mutants are outcompeted by RhlR cooperators during stationary phase. The competitive index of the RhlR-null mutant during co-culture with the RhlR cooperator is displayed on the *y*-axis as a function of time in hours. Data shown are the average and standard deviation of three biological replicates. In some cases, error bars are too small to be seen.

We reasoned that regularly monitoring the fitness of the RhlR-null mutant during co-culture with the parent E90 strain would offer additional insights regarding the mechanism(s) through which RhlR-null mutants are outcompeted. We measured the competitive index of the RhlR-null mutant every 2 hours starting at *t* = 4 hours. We found that the RhlR-null mutants are outcompeted sometime between 10 and 24 hours of co-culture (Fig. 5). Based on these data, we hypothesized that RhlR cooperators produce a factor during stationary phase that impairs or otherwise intoxicates RhlR mutants. To test this idea, we incubated E90 *rhlR* monocultures with cell-free supernatant harvested from the E90 overnight monoculture. However, we found that the E90-spent supernatant had no effect on the growth of the E90 *rhlR* mutant (Fig. S4B), suggesting that the factor is either cell surface-associated, volatile, highly reactive, or transiently produced.

## DISCUSSION

*P. aeruginosa* is an opportunistic pathogen that infects nearly 50% of adults with CF (26). Despite the importance of the QS regulator LasR in establishing infection, our group and others have demonstrated that LasR is commonly mutated in chronic *P. aeruginosa* CF isolates (15, 27, 28). In contrast to *lasR*, the coding sequence of *rhlR* is largely conserved, and we have shown that RhlR QS is maintained in many LasR-null isolates (15). These RhlR-active strains have been termed “RAIL,” for RhlR-active independent of LasR (16), and appear to be abundant in a variety of environmental settings. In addition, prior experimental evolution studies performed under conditions requiring QS for growth have also failed to observe mutations in *rhlR* (3, 4, 11). While others have demonstrated that RhlR mutants are rare because they are likely outcompeted by RhlR-active strains (8, 10, 11), relatively little is known about the myriad strategies used to maintain RhlR-mediated QS or cooperative behavior.

To understand how social dynamics may differ in LasR-null, RhlR-active, or “RhlR cooperator” backgrounds, we serially passaged a handful of clinical isolates individually in minimal medium with casein as the sole carbon source, which requires QS-regulated production of elastase for growth. In all of these genetic backgrounds, elastase production is dependent on RhlR. Consistent with the idea that inactivation of *rhlR* creates a cheating phenotype, in three out of four of these isolates RhlR-null mutants emerged in the population, and in two cases, came to a high percentage of the population (Fig. 1). Engineered RhlR-null mutants in the same genetic backgrounds had the same phenotype (Fig. 2), suggesting that cheating is due to mutation of *rhlR*, not an unrelated genetic change. These results also support the notion of RhlR-regulated policing (8, 29), as deletion of *rhlR* resulted in a significant fitness advantage (Fig. S2).

Interestingly, we found that RhlR-null cheaters were consistently unable to emerge in the well-characterized E90 background (19). To understand why RhlR-null mutants are heavily disfavored in E90, we studied the viability and relative fitness of an engineered, RhlR-null mutant. We found that RhlR-null mutants begin to be outcompeted by the parent strain during stationary phase due to an AHL QS-dependent mechanism. Currently, three mechanisms for maintaining QS in laboratory strains have been described: the RhlR-regulated production of and resistance to HCN, selective toxicity of phenazines, and prudent regulation of rhamnolipid production (8, 13, 30). However, we have shown that these strategies do not account for the phenotype we observed in the RhlR cooperator E90: mutants deficient in HCN or phenazine production are still able to outcompete the RhlR-null mutant (Fig. 4), and rhamnolipids are not required for the growth conditions of our experiments. Pleiotropic regulation of exoprotease and other non-cooperative products critical to E90 survival by RhlR could also yield the observed effects. Our previous transcriptomic analysis of the RhlR regulator in E90 and other LasR-null isolates found that while the core RhlR regulon may include as few as 20 genes (18, 19), the scope of E90 RhlR regulation includes an additional 33 genes, of which the function of many is still unknown. It is possible that a yet undetermined gene product regulated by QS in E90 confers the specific C4-HSL-RhlR-dependent cheater purging effect observed here. In an attempt to discern whether RhlR cooperators secrete a factor that intoxicates or otherwise impairs RhlR-null mutants, we assayed the growth of the RhlR-null mutant in the presence of E90-spent supernatant (Fig. 5). Although the E90-spent supernatant had no bactericidal or bacteriostatic effect on the RhlR-null mutant, it is possible that the factor(s) may be highly volatile and would lose activity during our preparation of the cell-free supernatant (31).

Our findings highlight the diversity of QS-mediated policing strategies, and their relative importance to controlling cheaters likely differs among isolates. While we have not identified the exact mechanism preventing RhlR-null mutants from emerging in the E90 background, our results nevertheless support the hypothesis that therapeutics targeting the RhlR-RhlI QS circuit may be beneficial in the context of a chronic infection. In theory, abrogating RhlR activity may destabilize populations of QS-proficient *P. aeruginosa* isolates and would presumably result in reduced expression of virulence factors. Recent research efforts have looked toward the design or discovery of small molecules that inhibit RhlR function (32, 33); however, the long-term effects of RhlR inhibition on the evolutionary trajectory of clinical isolates remain unclear. Investigating how the use of an RhlR inhibitor may affect social dynamics or the emergence of resistance to QS-based therapies would help clarify the viability of such an approach.

## MATERIALS AND METHODS

### Bacterial strains and growth conditions

We used either *Pseudomonas aeruginosa* PAO1 (34) or isolates that we have characterized genetically from the Early *Pseudomonas* Infection Control study (15, 18–20) (Table S1). Characteristics of the four EPIC isolates selected for study are presented in Table S2. Bacteria were grown in lysogeny broth buffered with 50 mM 3-(*N*-morpholino) propanesulfonic acid (LB-MOPS) at pH 6.8 or, alternatively, in a minimal medium called photosynthesis medium (PM) (35) with either 0.1% casamino acids (“CAA broth”) or 1% casein (“casein broth”) as the sole carbon source. Bacterial cultures were incubated in an orbital shaker at 37°C and 250 rpm unless otherwise specified.

### Growth curves

Bacterial strains and isolates were grown in 3 mL LB-MOPS for ~18 hours and inoculated at a 1:100 dilution into 3 mL fresh LB-MOPS until cultures had reached an optical density at 600 nm (OD600) of 0.2–0.5. Cultures were then inoculated into a 96-well plate at an OD600 of 0.01 and growth was monitored using a Biotek Synergy H1 plate reader by measuring the OD600 at 15-minute intervals. Growth rate was determined using the slope of the logarithmic phase of growth. Growth curves were performed in either LB-MOPS or CAA broth (Fig. S2).

### Fluorescent labeling of bacteria

Strains and isolates were labeled with a chromosomal copy of *gfp*, or in the case of E90, *mCherry*, using the mini-Tn7 system as previously described (36). Briefly, strains and isolates were grown overnight and triparentally mated with *Escherichia coli* containing a plasmid with either the Tn7 integration machinery or *E. coli* containing a plasmid with *gfp* and a gentamicin-resistance marker (Table S1). Bacteria were plated on LB agar containing 100 µg/mL gentamicin, and GFP fluorescence was confirmed by using a Biotek Synergy H1 plate reader (489 nm excitation, 520 nm emission for GFP; 587 nm excitation, 620 nm emission for mCherry).

### Casein competitions

Wild-type and *rhlR* mutant strains, as well as PAO1 and PAO1 *lasR,* were individually inoculated into 3 mL of LB-MOPS and grown overnight. Casein broth was then inoculated with 200 µL of the parent strain and 20 µL *rhlR* mutant in 4 mL of casein broth. Cultures were passaged into fresh media (200 µL into 4 mL casein broth) at 24-hour intervals and sampled at the same timepoints. Cultures were plated to identify individual colonies. Frequencies of *rhlR* mutants and the parent strains were determined by identification of zones of proteolysis on skim milk plates, as previously described (4). Alternatively, when *rhlR* mutants were *gfp*-labeled, cultures were analyzed using a BD Accuri C6+ flow cytometer to enumerate fluorescence of 10,000 cells per sample and determine the ratio of wild-type individuals to *rhlR* mutants. Because proteolyzed casein can cause blockage in the cytometer, cultures were transferred to fresh LB-MOPS and grown for 7 hours before analysis by flow cytometry.

### *In vitro* evolution

To evaluate the emergence of protease-negative mutants, parent strains were grown in casein broth and serially passaged as previously described (4). Briefly, cultures were grown overnight in LB-MOPS, and 200 µL of this overnight culture were inoculated into 4 mL casein broth. Cultures were passaged to fresh casein broth initially at 48-hour intervals, and subsequently at 24-hour intervals once growth was sufficiently robust. Colony-forming units from each culture were determined at the time of passage by dilution plating, and the frequency of protease-negative isolates was determined by plating on skim milk agar, as previously described. We sequenced *rhlR* from protease-negative colonies (and a selection of protease-producing colonies) using primers rhlRAlleConf_SeqF (5′-CCAGCACACACATGAGGGG-3′) and rhlRAlleConf_SeqR (5′-GGCTGCGTCCTGAACGGTG-3′).

## SUPPLEMENTAL MATERIAL

Figure S1 jb.00344-24-s0001.pdf Selected RhlR-null mutants have a differential ability to grow on casein.

Figure S2 jb.00344-24-s0002.pdf Competitive indices of 6 wild-type and RhlR-null mutant pairs show no consistent increase in RhlR-null mutant frequency.

Figure S3 jb.00344-24-s0003.pdf The E90 RhlR-null mutant grows comparably to the parent strain in casamino acids broth.

Figure S4 jb.00344-24-s0004.pdf RhlR mutants of E90 are outcompeted by the parent, but do not grow differently in E90 spent supernatant.

Table S1 jb.00344-24-s0005.pdf Bacterial strains, plasmids, and primers used in this study.

Table S2 jb.00344-24-s0006.pdf Features of EPIC isolates used in this study.

ASM does not own the copyrights to Supplemental Material that may be linked to, or accessed through, an article. The authors have granted ASM a non-exclusive, world-wide license to publish the Supplemental Material files. Please contact the corresponding author directly for reuse.
