# Supplementary material for: RhlR-mediated cooperation in cystic fibrosis-adapted isolates of Pseudomonas aeruginosa
Source: J Bacteriol. 2024 Dec 13;207(1):e00344-24. doi: 10.1128/jb.00344-24 (PMC11784195; doi:10.1128/jb.00344-24)
Supplement: Figure S1 — Selected RhlR-null mutants have a differential ability to grow on casein. [file jb.00344-24-s0001.pdf]

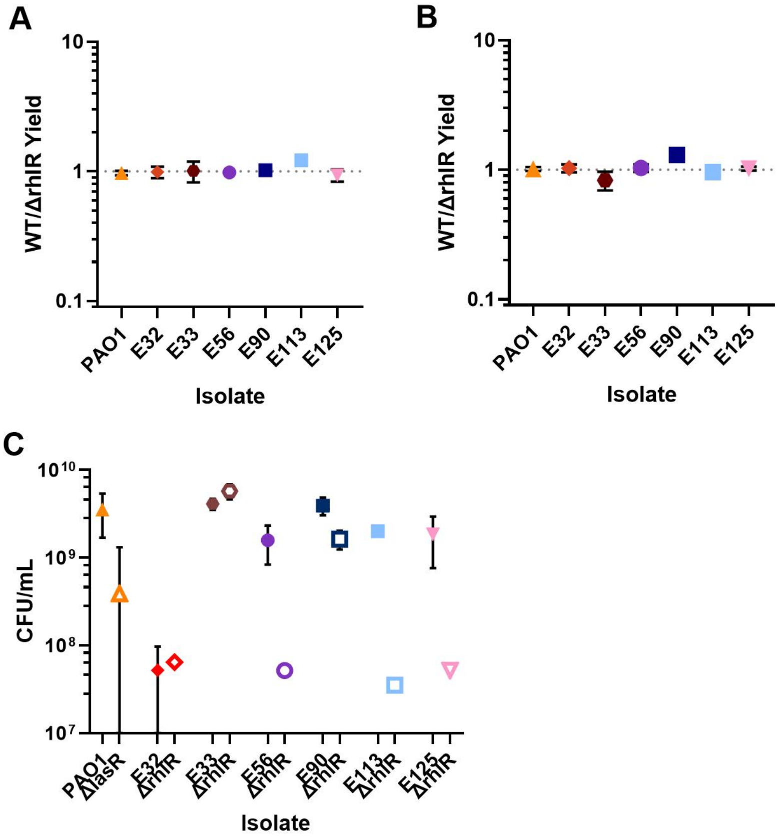

Supplemental Figure 1. Selected RhlR-null mutants have a differential ability to grow on casein. Relative growth yield of the parent wild-type clinical strains versus their isogenic RhlR-null mutants in (A) buffered LB or (B) minimal media supplemented with 1% casamino acids. A relative growth yield of 1 indicates that the wild-type and mutant strains grow equally well in the respective media, while <1 indicates the RhlR-null mutant has a lower yield and >1 indicates a higher yield. (C) CFU/mL of wild-type and RhlR-null mutants at 24 hours of growth in casein broth. The differences between the parent and RhlR-null mutant are all significantly different by pairwise t-test ($p < 0.05$) except E32 and E33.
