# Supplementary material for: RhlR-mediated cooperation in cystic fibrosis-adapted isolates of Pseudomonas aeruginosa
Source: J Bacteriol. 2024 Dec 13;207(1):e00344-24. doi: 10.1128/jb.00344-24 (PMC11784195; doi:10.1128/jb.00344-24)
Supplement: Figure S2 — Competitive indices of 6 wild-type and RhlR-null mutant pairs show no consistent increase in RhlR-null mutant frequency. [file jb.00344-24-s0002.pdf]

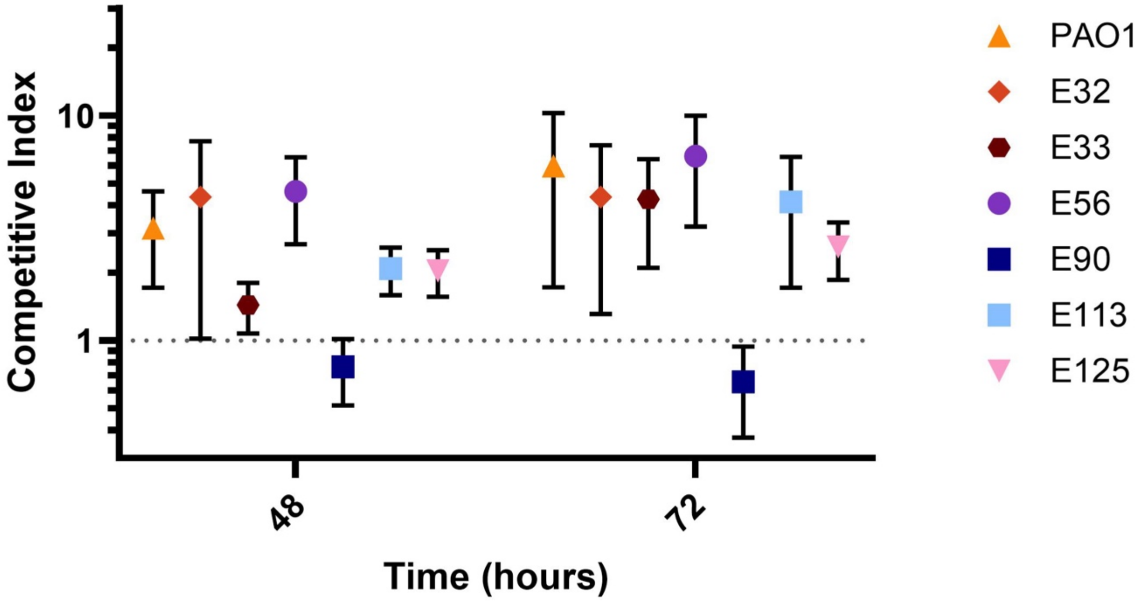

Supplemental Figure 2. Competitive indices of 6 wild-type and RhlR-null mutant pairs show no consistent increase in RhlR-null mutant frequency. E32, E33, E56, E90, E113, and E125 strains were inoculated in co-culture with their wild-type parent in 1% casein at an initial frequency of 10% RhlR-null mutants and serially passaged every 24 hours. The frequency of the protease-deficient RhlR-null mutant phenotype within the co-culture was assessed using skim milk plates.
