# Supplementary material for: RhlR-mediated cooperation in cystic fibrosis-adapted isolates of Pseudomonas aeruginosa
Source: J Bacteriol. 2024 Dec 13;207(1):e00344-24. doi: 10.1128/jb.00344-24 (PMC11784195; doi:10.1128/jb.00344-24)
Supplement: Figure S3 — The E90 RhlR-null mutant grows comparably to the parent strain in casamino acids broth. [file jb.00344-24-s0003.pdf]

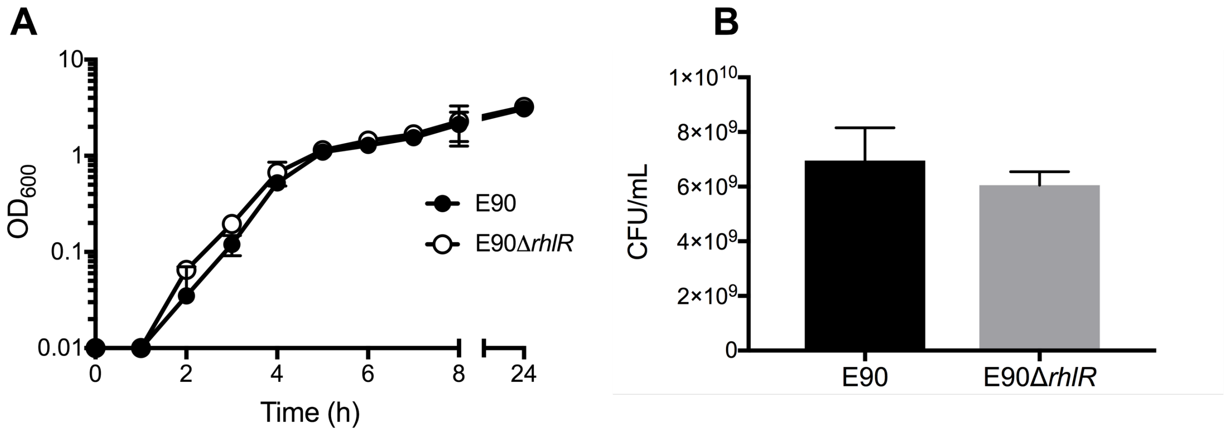

Supplemental Figure 3. The E90 RhlR-null mutant grows comparably to the parent strain in casamino acids broth. A) Growth curve in minimal casamino acids in 18 mm culture tubes. B) Cell yield following 24 h incubation. Data shown are the average and standard deviation of three biological replicates. In some cases, error bars are too small to be seen.
