# Supplementary material for: RhlR-mediated cooperation in cystic fibrosis-adapted isolates of Pseudomonas aeruginosa
Source: J Bacteriol. 2024 Dec 13;207(1):e00344-24. doi: 10.1128/jb.00344-24 (PMC11784195; doi:10.1128/jb.00344-24)
Supplement: Figure S4 — RhlR mutants of E90 are outcompeted by the parent, but do not grow differently in E90 spent supernatant. [file jb.00344-24-s0004.pdf]

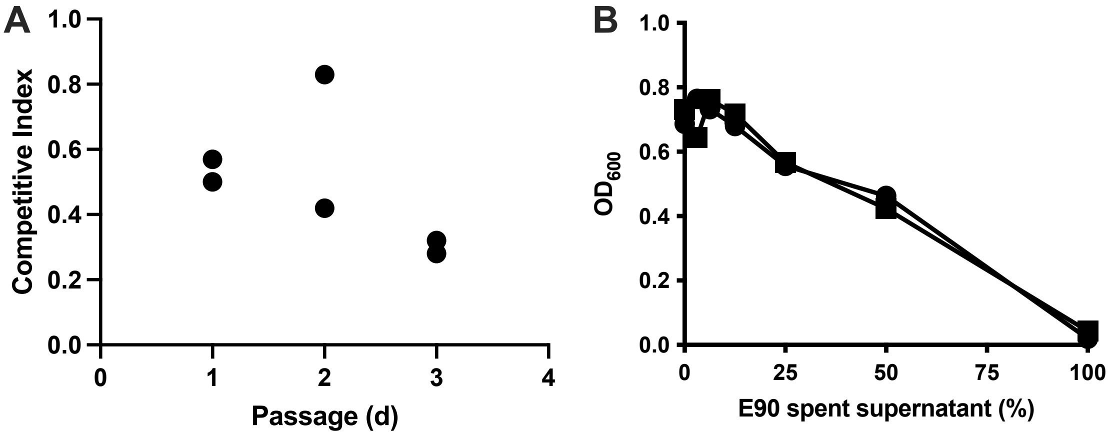

Supplemental Figure 4. RhlR mutants of E90 are outcompeted by the parent, but do not grow differently in E90 spent supernatant. (A) Competitive index of RhlR mutants when grown in co-culture with E90, as measured by patching inidivudal isolates on skim milk agar. (B). Optical density after 18h overnight growth in spent supernatant from E90 grown in casamino acids, supplemented with fresh media. Closed squares: E90; Closed circles: E90Δ*rhlR*
