# Supplementary material for: RhlR-mediated cooperation in cystic fibrosis-adapted isolates of Pseudomonas aeruginosa
Source: J Bacteriol. 2024 Dec 13;207(1):e00344-24. doi: 10.1128/jb.00344-24 (PMC11784195; doi:10.1128/jb.00344-24)
Supplement: Table S1 — Bacterial strains, plasmids, and primers used in this study. [file jb.00344-24-s0005.pdf]

Table S1. Bacterial strains, plasmids, and primers used in this study.

Bacterial strains	Description	Reference
P. aeruginosa strains		
PAO1	Wild-type laboratory strain	1
PAO1 Δ*lasR*	PAO1 derivative "DA5" with unmarked, in-frame *lasR* deletion	2
E32	Chronic infection isolate from pediatric CF patient; has a non-synonymous mutation (C649T) in *lasR*	3
E32 Δ*rhlR*	E32 containing an unmarked, in-frame *rhlR* deletion from residues 3-240	4
E33	Chronic infection isolate from pediatric CF patient; has a non-synonymous mutation (G61A) in *lasR*	3
E33 Δ*rhlR*	E33 containing an unmarked, in-frame *rhlR* deletion from residues 3-240	4
E41	Chronic infection isolate from pediatric CF patient; has a nonsense mutation (G31T) in *lasR*	3
E41 Δ*rhlR*	E41 containing an unmarked, in-frame *rhlR* deletion from residues 3-240	4
E56	Chronic infection isolate from pediatric CF patient; has a non-synonymous mutation (G338A) in *lasR*	3
E56 Δ*rhlR*	E56 containing an unmarked, in-frame *rhlR* deletion from residues 3-240	4
E56 Δ*rhlR-gfp*	E56 Δ*rhlR* marked with chromosomally integrated *gfp* inserted at a neutral site; Gm^R	This study
E86	Chronic infection isolate from pediatric CF patient; has an 11-bp deletion at nt 97 (-TCGAAGATCCT) relative to the coding sequence start in *lasR*	3
E86 Δ*rhlR*	E86 containing an unmarked, in-frame *rhlR* deletion from residues 3-240	4
E90	Chronic infection isolate from pediatric CF patient; has a 1-bp deletion at nt 170 (-C) relative to the coding sequence start in *lasR*	3
E90 Δ*rhlR*	E90 containing an unmarked, in-frame *rhlR* deletion from residues 3-240	5
E90 Δ*rhlR*-mCherry	E90 Δ*rhlR* marked with chromosomally integrated mCherry inserted at a neutral site; Gm^R	This study
E90 Δ*rhlI*	E90 containing an unmarked, in-frame *rhlI* deletion	5[a]
E104	Chronic infection isolate from pediatric CF patient; has a non-synonymous mutation (A532G) in *lasR*	3
E104 Δ*rhlR*	E104 containing an unmarked, in-frame *rhlR* deletion from residues 3-240	4
E113	Chronic infection isolate from pediatric CF patient; has a non-synonymous mutation (T55C) in *lasR*	3
E113 Δ*rhlR*	E113 containing an unmarked, in-frame *rhlR* deletion from residues 3-240	4
E113 Δ*rhlR-gfp*	E113 Δ*rhlR* marked with chromosomally integrated *gfp* inserted at a neutral site; Gm^R	This study

E125	Chronic infection isolate from pediatric CF patient; has a nonsense mutation (C280T) in *lasR*	3
E125 ∆*rhlR*	E125 containing an unmarked, in-frame *rhlR* deletion from residues 3-240	4
E125 ∆*rhlR-gfp*	E125 ∆*rhlR* marked with chromosomally integrated *gfp* inserted at a neutral site; Gm^R	This study
E129	Chronic infection isolate from pediatric CF patient; has a non-synonymous mutation (C149T) in *lasR*	3
E129 ∆*rhlR*	E129 containing an unmarked, in-frame *rhlR* deletion from residues 3-240	4
E131	Chronic infection isolate from pediatric CF patient; has a non-synonymous mutation (A580G) in *lasR*	3
E131 ∆*rhlR*	E131 containing an unmarked, in-frame *rhlR* deletion from residues 3-240	4
E167	Chronic infection isolate from pediatric CF patient; has a 1-bp deletion at nt 339 (-G) relative to coding sequence start in *lasR*	3
E167 ∆*rhlR*	E167 containing an unmarked, in-frame rhlR deletion from residues 3-240	4
E. coli strains		
BW29427	Donor strain used in triparental mating harboring mini-Tn7-based vectors; diaminopimelic acid (DAP) auxotroph; Genotype *thrB1004 pro thi rpsL hsdS lacZ* ΔM15 *RP4-1360* Δ(*araBAD*)*567* Δ*dapA1341*::[*erm pir*]*tra*	6
ß2155	Helper strain used in triparental mating harboring pUX-BF13; Genotype: *thrB1004 pro thi strA hsdsS lacZΔM15 (F' lacZΔM15* $lacI^q$ *traD36* $proAB^+$*) ΔdapA::erm(*Ery^R*) pir::RP4* [::kan (Kan^R) from SM10]	7

**Plasmids**	**Description**	**Reference**
pUC18-mini-Tn7-Gm-GFP	mini-Tn7-based vector for chromosomal integration of *gfp* and gentamicin resistance cassette at neutral *att* site; Gm^R	8
pUC18-mini-Tn7-Gm-mCherry	mini-Tn7-based vector for chromosomal integration of mCherry and gentamicin resistance cassette at neutral *att* site; Gm^R	9
pUX-BF13	R6K replicon-based helper plasmid providing the Tn7 transposon genes; mob^+; Ap^R	10

a. E90 ∆rhlI was first reported in Cruz et al, 2000 (5) but inadvertently left out of the “bacterial strains and plasmids” table in that manuscript
