# Supplementary material for: RhlR-mediated cooperation in cystic fibrosis-adapted isolates of Pseudomonas aeruginosa
Source: J Bacteriol. 2024 Dec 13;207(1):e00344-24. doi: 10.1128/jb.00344-24 (PMC11784195; doi:10.1128/jb.00344-24)
Supplement: Table S2 — Features of EPIC isolates used in this study. [file jb.00344-24-s0006.pdf]

Table S2. Features of EPIC isolates used in this study.

Isolate	*lasR* mutation type	*lasR* mutation	Protease phenotype[a]	LasR signal, μM[b] (3OC12-HSL)	RhlR signal, μM (C4-HSL)
E56	Non-synonymous	G338>A	Moderate	0.181	10.467
E90	Deletion	170delC(fs)	Moderate	0.055	7.149
E113	Non-synonymous	T55>C	High	0.045	3.445
E125	Non-synonymous	C280>T	Low	0.068	13.429

[a] Qualitative assessment of elastase produced by each strain after growth on skim milk agar, as compared to wildtype PAO1.

[b] Measurement of 3OC12-HSL or C4-HSL produced after overnight growth of each strain on LB-MOPS, as determined by using bioreporter assays (11, 12)**.**

Supplemental references

1. Stover CK, Pham XQ, Erwin AL, Mizoguchi SD, Warrener P, Hickey MJ, Brinkman FS, Hufnagle WO, Kowalik DJ, Lagrou M, Garber RL, Goltry L, Tolentino E, Westbrock-Wadman S, Yuan Y, Brody LL, Coulter SN, Folger KR, Kas A, Larbig K, Lim R, Smith K, Spencer D, Wong GK, Wu Z, Paulsen IT, Reizer J, Saier MH, Hancock RE, Lory S, Olson MV. 2000. Complete genome sequence of *Pseudomonas aeruginosa* PAO1, an opportunistic pathogen. Nature 406:959-64.
2. Wang M, Schaefer AL, Dandekar AA, Greenberg EP. 2015. Quorum sensing and policing of *Pseudomonas aeruginosa* social cheaters. Proceedings of the National Academy of Sciences of the United States of America 112:2187-91.
3. Feltner JB, Wolter DJ, Pope CE, Groleau MC, Smalley NE, Greenberg EP, Mayer-Hamblett N, Burns J, Deziel E, Hoffman LR, Dandekar AA. 2016. LasR variant cystic fibrosis isolates reveal an adaptable quorum sensing hierarchy in *Pseudomonas aeruginosa*. mBio 7:e01513-16.
4. Asfahl KL, Smalley NE, Chang AP, Dandekar AA. 2022. Genetic and transcriptomic characteristics of RhlR-dependent quorum sensing in cystic fibrosis isolates of *Pseudomonas aeruginosa*. mSystems 7:e0011322.
5. Cruz RL, Asfahl KL, Van den Bossche S, Coenye T, Crabbe A, Dandekar AA. 2020. RhlR-regulated acyl-homoserine lactone quorum sensing in a cystic fibrosis isolate of *Pseudomonas aeruginosa*. mBio 11.
6. Lee HH, Ostrov N, Wong BG, Gold MA, Khalil AS, Church GM. 2019. Functional genomics of the rapidly replicating bacterium *Vibrio natriegens* by CRISPRi. Nat Microbiol 4:1105-1113.
7. Dehio C, Meyer M. 1997. Maintenance of broad-host-range incompatibility group P and group Q plasmids and transposition of Tn5 in *Bartonella henselae* following conjugal plasmid transfer from *Escherichia coli*. J Bacteriol 179:538-40.
8. Choi KH, Schweizer HP. 2006. mini-Tn7 insertion in bacteria with single attTn7 sites: example *Pseudomonas aeruginosa*. Nat Protoc 1:153-61.
9. Zhou H, Wang M, Smalley NE, Kostylev M, Schaefer AL, Greenberg EP, Dandekar AA, Xu F. 2019. Modulation of *Pseudomonas aeruginosa* quorum sensing by glutathione. J Bacteriol 201.
10. Bao Y, Lies DP, Fu H, Roberts GP. 1991. An improved Tn7-based system for the single-copy insertion of cloned genes into chromosomes of gram-negative bacteria. Gene 109:167-8.

11. Chugani SA, Whiteley M, Lee KM, D'Argenio D, Manoil C, Greenberg EP. 2001. QscR, a modulator of quorum-sensing signal synthesis and virulence in *Pseudomonas aeruginosa*. Proc Natl Acad Sci U S A 98:2752-7.
12. Lee JH, Lequette Y, Greenberg EP. 2006. *Activity of purified Q*scR, a Pseudomonas aeruginosa orphan quorum-sensing transcription factor. Mol Microbiol 59:602-609.
